# Evaluation of Nutritional Status of Elderly Patients Presenting to the Family Health Center

**DOI:** 10.12669/pjms.342.14936

**Published:** 2018

**Authors:** Son Nazan, Kavak Buket

**Affiliations:** 1 Son Nazan, Faculty of Medicine, History of Medicine and Ethics Department Head, Afyon Health School, Nutrition and Dietetic Department Head, Afyon Kocatepe University, Afyonkarahisar, Turkey; 2 Kavak Buket, Free Dietitian, Afyonkarahisar, Turkey

**Keywords:** Elderly, Malnutrition, Mini Nutritional Assessment (MNA)

## Abstract

**Objective:**

Nutritional problems are one of the most common disorders encountered in aging, and malnutrition, secondary to insufficient food consumption. Failure to diagnose malnutrition can increase the risk of illnesses and death. Our objective was to evaluate the nutritional status of elderly patients aged 65 years and older through a Mini Nutritional Assessment (MNA) scale and to determine its relationship with laboratory parameters.

**Methods:**

This is a single-center cross-sectional study which included 102 patients aged 65 years and older who presented to the Family Health Center (FHS) in the Cobanlar District in the Province of Afyon, Turkey. Anthropometric and biochemical measurements of the patients were obtained, and the MNA scale was administered to the patients using a face-to-face interview technique. In the statistical analysis of the data, categorical variables were analyzed using a Chi-square test, normally distributed data were analyzed using an analysis of variance (ANOVA), continuous variables with normal distribution comprising independent measurements were analyzed using an Independent Samples T-test, and data without normal distribution were analyzed using a Mann-Whitney Rank Sum test.

**Results:**

Of the 102 patients included in this study, 45 (44.1%) were male and 57 (55.9%) were female. The mean age was 74.06±7.89 years (min-max: 65–92). Of these patients, 39 (38.2%) were found to have malnutrition, 19 (18.6%) were at high risk of malnutrition and 44 (43.1%) had a normal nutritional status.

**Conclusions:**

The findings suggest evaluating nutritional status in individuals aged 65 years and older in the population on a regular basis to reduce disease risk and mortality.

## INTRODUCTION

Malnutrition, in simple terms, refers to the lack of nutrition and is characterized by the inability to consume the necessary nutrients required by the body.^1^ The European Association for Clinical Nutrition and Metabolism (ESPEN) has reported an increased risk of malnutrition worldwide^2^, and while it can be observed in different groups within the population, it is particularly prevalent among the elderly. Malnutrition and risks of developing malnutrition in the elderly may have various negative results, such as prolonging hospital stays, increasing healthcare costs, decreasing quality of life and increasing the risk of death.^3^

Life expectancy has increased in parallel to the improvements in the quality of life and the decrease in the birth rates worldwide, and this has resulted in a gradual increase in the elderly population. According to the expectations of the World Health Organization, it is estimated that there will be approximately 1.2 billion elderly people in 2025, and this figure will reach two billion by 2050, with 80% of the elderly population in the world living in developing countries.^4^

Elderly people (aged 65 years and older) made up 7.7% of the global population in 2013, and it is estimated that this will have increased to 10.2% by 2023, to 20.8% by 2050 and 27.7% by 2075.^5^

Changes in food consumption occur as people advance in age. In the elderly, inadequate food consumption due to a loss of appetite results in a decrease in muscle and fat mass and the development of malnutrition.^6^

Keeping a daily food log is extremely important in detecting conditions that develop secondary to nutritional status, although keeping such a food log can be difficult, which has led to the development of various screening tests for the measurement of nutritional status.

The Mini Nutritional Assessment (MNA) scale developed by Guigoz et al. in 1994 has been used as a screening test for the detection of malnutrition.^7^ European guidelines on nutritional screening recommend the use of MNA or the Mini Nutritional Assessment Short- Form (MNA-SF) in the geriatric population.^8^ In a report published in 2002, ESPEN stated that all individuals aged 65 years and older must be screened for their nutritional status at certain intervals, and similar recommendations are made in other ESPEN guidelines published since 2002.^9^ According to ESPEN, malnutrition is clinically defined as the inadequate consumption of food. However, malnutrition also reflects physical changes and the consequences of changes in function.^10^ Malnutrition causes impairments in physical and mental functioning, and the findings showed that providing nutritional support to patients with malnutrition leads to a restoration of physical and mental functioning.^11^ Nutritional screening is required to provide nutritional support.

There are various studies of MNA and MNA-SF in different parts of the world. In a study by Kaiser et al. which aimed to detect cases of malnutrition in the community, MNA was used as a screening tool, and different rates were reported for the elderly people living in nursing homes, hospitals and in the community. The authors identified a correlation between malnutrition and dementia and sarcopenia and suggested that the MNA could be used to evaluate the nutritional status of elderly people.^12^ Another study conducted on the geriatric population using the MNA scale found that individuals with malnutrition enter the aging process more rapidly, and have higher mortality rates than individuals with normal nutritional status.^13^ Similarly, in another study, the MNA scale was administered to inpatients that had suffered heart failure and found that these patients had a higher mortality rate.^14^ The present study aims to detect the prevalence of malnutrition in the elderly Turkish population using the MNA scale and to compare the results with the individuals in the literature.

## METHODS

This single-center cross-sectional study was conducted at a Family Health Center (FHC) located in the Cobanlar District of the Province of Afyonkarahisar in Turkey. Individuals aged 65 years and older living in the district of Cobanlar were included in the study. Approximately 1,000 people aged 65 years and above resided in the district of Cobanlar, and around 120 individuals aged 65 years and older are admitted per month to the FHC. The study sample comprised of 102 patients who were admitted to the FHC between June 15, 2017 and August 1, 2017, who agreed to take part in the survey, and who had no severe psychiatric condition, dementia or communication problems (i.e. severe hearing and visual impairment). This study was approved by the Head Physician’s Office at the R.T Cobanlar District Hospital on June 1, 2017 with (23252106-000-67) and by the Okan University Clinical Ethics Committee on June 5, 2017 with decree number 84.

The participants who met the study inclusion criteria were provided with general information about this study, and, sociodemographic data were generated through face-to-face interviews. Anthropometric measurements were made, and the data were recorded; information on health status was inquired, and biochemical parameters from the last one month were retrieved from the archive. The MNA questionnaire was administered to the participants, with an additional 13 questions asked to garner sociodemographic, anthropometric and general health data, including age, gender, educational status, household, height, weight, waist and hip circumference, dependency on bed, presence and identity of chronic diseases, blood pressure, total blood protein levels and fasting blood glucose. The MNA investigated anthropometric measurements (BMI, mid-arm circumference [MAC], calf circumference and body weight), diet changes (number of meals, food and fluid intake, independent eating), overall assessment parameters (lifestyle, medications used, mobility, stress, dementia or depression status) and personal assessment parameters (self-assessment on own health status and nutritional status).

The questionnaire took approximately 5–10 minutes to complete, with height (cm) using a fixed height scale, weight (kg) using a fixed weight scale, blood pressure, waist circumference using a tape, hip circumference, mid-upper arm circumference and calf circumference all measured by the same person. Fasting blood glucose and blood protein levels were retrieved from measurements form over the last one-month period recorded on the patient’s chart.

The validity and reliability of the MNA were evaluated by Guigoz et al. in 1994 and was deemed appropriate for the evaluation of malnutrition. A further three studies, two in France (155 and 120 patients) and one in New Mexico (347 patients) also validated the tool, which is applied in two stages. MNA-SF scale is administered in the first stage, and if the scores indicate risk of malnutrition, the MNA scale is administered in the second stage. The validity and reliability of the MNA and MNA-SF have been evaluated by Dr. Derya Sarikaya in Turkey, and are used as a screening tool for malnutrition in elderly patients.^15^

The MNA comprises 18 questions, including 15 verbal questions and three questions based on anthropometric measurement. The scoring is performed on a 30-point scale, and the questionnaire starts with six screening questions yielding a maximum of 14 points (MNA-SF). These six questions (A-F) evaluate appetite, weight loss in recent months, mobility, psychological stress or acute disease, dementia or depression status, and BMI.^7^ After administering the first section of the test, if the nutritional status was found to be normal, i.e. if the total score is ≥12 (maximum 14 points), the questionnaire is terminated. If the subtotal score is ≤12, it is considered that malnutrition exists, and the interview proceeds with other questions (G-S) (MNA). These questions relate to place of residence, medication use, pressure or skin ulcers or presence of skin inflammation, the number of daily meals, protein and fluid consumption, mode of feeding, self-view of the patient about nutritional and health status, and some additional anthropometric measurements.^16^,^17^ A total MNA score of ≤17 indicates the presence of malnutrition, a score of 17–23 indicates malnutrition risk, and a score of ≥23.5 indicates normal nutritional status.^18^

### Data Analysis and Statistical Methods

The continuous quantitative variables were expressed as number, mean and standard deviation; and the qualitative or score variables were expressed as number, median, 25th and 75th percentile. Continuous variables with a normal distribution of independent measurements were analyzed using an Independent Samples T-test, and data without normal distribution were analyzed using a Mann-Whitney Rank Sum Test. A one-way ANOVA was used for continuous variables, and a Pearson Chi-square test was used for categorical variables. A probability of P<0.05 was considered statistically significant, and all data were analyzed using the SPSS 21 software package.

## RESULTS

The study sample included 45 (44.1%) men and 57 (55.9%) women aged ≥65 years (mean= 74.06 years), who were residing in Cobanlar in Afyonkarahisar, located in western Turkey. The characteristics of the study participants are shown in Table-I. The mean age was comparable between the male (77.2±8.4) and female (77.2±8.6) patients. Of the total, 53 (52%) were illiterate, 23 (22.5%) were literate, 25 (24.5%) were primary school graduates, and one (1%) was a high-school graduate.

**Table-I:** Characteristics of the study participants.

Characteristics	Malnutrition (n=39) (women=24, men= 15)	Malnutrition risk (n=19) (women=10, men= 9)	Normal nutrition (n=44) (women=23, men= 21)	p
***Age (years)***				
Women	79.21±9.29^d^	72.36±4.37^b^	70.32±4.13^d^	
	77.50 (71.25-88.25)^a^	72.00 (70.00-77.00)^c^	70.00 (67.00-74.00)^a^	
	0.707^p^	0.517^p^	0.246^p^	
Men	81.07±7.91^d^	71.00±4.85^b^	69.33±4.68^d^	
	83.00 (77.00-87.00)^a^	71.00 (66.00-75.00)^c^	68.00 (65.50-73.00)^a^	
	0.707^p^	0.517^p^	0.246^p^	
***Education level***				
No Education	29 (54.7)	6 (11.3)	18 (34)	0.010
Only read and write	6 (26.1)	6 (26.1)	11(47.8)	0.010
Primary school	4 (16)	6 (24)	15 (60)	0.010
High school	0 (0)	1 (100)	0 (0)	0.010
***Comorbidities***				
Diabetes	12 (31.6)	4 (10.5)	22 (57.9)	0.053
Hypertension	22 (46.8)	10 (21.3)	15 (31.9)	0.296
Hypoproteinemia	36 (72)	9 (18)	5 (10)	0.001
Others	16 (47)	6 (17.6)	12 (35.4)	0.035
***Place of residence***				
Family	21 (26.3)	18 (22.5)	41 (51.3)	0.001
Single	18 (81.8)	1 (4.5)	3 (13.6)	0.001
***Nutritional status***				
Severe anorexia	29 (87.9)	1 (3)	3 (9.1)	0.001
Mild anorexia	10 (33.3)	14 (46.7)	6 (20)	0.001
Normal (regular)	0 (0)	4 (10.3)	35 (89.7)	0.001
***BMI (kg/m^2^)***				
Women	21.79±3.67^d^	27.59±4.30^b^	31.86±6.85^d^	
	21.06 (19.65-22.79)^a^	26.91 (24.49-31.11)^c^	31.59 (25.85-35.66)^a^	
	0.149^p^	0.439^p^	0.296^p^	
Men	20.26±1.96^d^	26.33±2.25^b^	29.48±4.50^d^	
	19.96 (18.51-21.79)^a^	26.67 (24.75-27.91)^c^	27.72 (26.34-31.45)^a^	
	0.149^p^	0.439^p^	0.296^p^	
***MAC (cm)***				
Women	20.96±3.34^d^	27.09±4.89^b^	27.56±2.46^d^	
	20.25 (18.00-23.00)^a^	25.00 (24.00-30.00)^c^	25.00 (24.00-29.50)^a^	
	0.695^p^	0.841^p^	<0.001^p^	
Men	21.27±2.50^d^	26.67±4.30^b^	30.10±3.00^d^	
	21.50 (19.00-22.00)^a^	25.00 (23.50-30.50)^c^	29.00 (27.00-31.45)^a^	
	0.695^p^	0.841^p^	<0.001^p^	
***CC (cm)***				
Women	30.23±4.44^d^	32.96±0.97^b^	38.32±7.79^d^	
	30.00 (27.50-32.75)^a^	33.00 (32.15-33.63)^c^	36.00 (32.50-42.00)^a^	
	0.541^p^	<0.001^p^	0.946^p^	
Men	30.77±3.16^d^	36.48±2.12^b^	38.56±7.55^d^	
	30.00 (29.00-32.00)^a^	36.00 (34.75-38.00)^c^	37.00 (32.50-46.00)^a^	
	0.541^p^	<0.001^p^	0.946^p^	
MNA Scores	11.64±3.224	20.30±1.551	26.99±2.814	

Baseline characteristics of the participants. Data presented as number (percent) for the following variables: educational level, specific comorbidities, place of residence and nutritional status. Median (25%–75%)^a^Mean±Std. Deviation^b^, Independent Samples T-Test^c^, Mann-Whitney Rank Sum Test^d^For other variables, the mean±SD are used. One-way ANOVA was used for continuous variables, and the Pearson chi-squared test was used for categorical variables. During testing, p<0.05 was considered statistically significant. BMI: body mass index; CC: calf circumference; MAC: mid-arm circumference; MNA: Mini Nutritional Assessment. P-value ^p^.

Regarding residential status, 22 patients (21.6%) lived alone and 80 (78.4%) lived with their families, while 81 (79.4%) patients were independent, 15 (14.7%) were semi-dependent and six (5.9%) were bed bound.

## DISCUSSION

There are many causes of malnutrition in the geriatric population, and its early diagnosis and treatment can improve the prognosis of existing diseases in the elderly. Malnutrition in the elderly population requires a multidisciplinary approach involving both social aspects and interventions into eating habits and is more common among inpatients and homecare patients.^19^

In a cross-sectional study conducted by Saka et al., 413 elderly patients presenting at outpatient clinics in the preceding 12-month period were evaluated with an MNA test, and findings showed that 13% of the study sample were malnourished, and 31% were at risk of malnutrition. Furthermore, the findings showed that patients with malnutrition and patients at risk of developing malnutrition had lower hemoglobin, serum total protein and albumin levels than the normal population, and comorbidities commonly accompanied malnutrition.^20^ The present study had similar findings, with statistical significance (Table-I).

Guigoz et al. found out that the rate of malnutrition is higher among inpatients, nursing home residents and patients living along^16^, while in the present study, the rate of malnutrition is higher among the elderly living alone than in the normal population.

In a study by Feldblum involving 204 patients, the findings showed that males are at a higher risk of developing malnutrition than females and that the risk of malnutrition increases with age.^21^ In a study asserting the contrary and involving 152 elderly patients, the rate of malnutrition is reported as 5.3%, and patients at risk of malnutrition stood at 32.9%. It is found further in the study that the prevalence of malnutrition is higher in females than in males, as well as in older patients, and patients with malnutrition have poorer appetites.^22^ Similar results were found in the present study. Patients with malnutrition displayed a severe lack of appetite, the malnutrition risk group had a poor appetite, and there were statistically significant differences between the groups when compared with patients of normal nutritional status.

BMI, CC and MAC values have an important place in the diagnosis of malnutrition. In a study in China, X. Hu and et al. used the MNA test, and corroborating the results of the present study, they found the lowest BMI, CC and MAC values in the malnutrition group, that the values in the risk group were slightly higher, and that normal population recorded the highest values in both genders.^23^ The present study found that the risk of malnutrition increases with age in the geriatric population, that diseases accompanying malnutrition increase and BMI is higher in females, that females have lower CC and MAC values than males, BMI, CC, MAC and blood protein levels decrease with increasing severity of malnutrition, and the risk of malnutrition is higher in the elderly population. Although the study sample represents only a small proportion of Turkey’s geriatric population, the sample does represent the population in the region. Further studies may be conducted on a larger sample by involving different regions.

## CONCLUSION

The rate of malnutrition is rapidly increasing in parallel with the growth of the global elderly population. As malnutrition increases mortality, the use of nutritional screening tests among the elderly population would support the application of health measures to be taken in this direction.
